# *Moringa oleifera* Seeds Attenuate Vascular Oxidative and Nitrosative Stresses in Spontaneously Hypertensive Rats

**DOI:** 10.1155/2017/4129459

**Published:** 2017-06-20

**Authors:** Joseph Iharinjaka Randriamboavonjy, Marc Rio, Pierre Pacaud, Gervaise Loirand, Angela Tesse

**Affiliations:** ^1^INSERM, CNRS, Université de Nantes, l'institut du thorax, 44000 Nantes, France; ^2^CHU de Nantes, 44000 Nantes, France

## Abstract

*Moringa oleifera* (MOI) is a tree currently used in traditional medicine in tropical Africa, America, and Asia for therapeutic applications in several disorders including arterial hypertension. We previously described a cardiac protective role of a treatment with MOI seeds in spontaneously hypertensive rats (SHR). Here, we investigated the effects of this treatment on oxidative and nitrosative vascular stresses in SHR, with normotensive Wistar Kyoto rats used as controls. Oxidative and nitrosative stresses detected in SHR aortas using the fluorescent dye dihydroethidine and protein nitrotyrosine staining were reduced in MOI-treated SHR aortas. This was associated with a decrease of free 8-isoprostane circulating level, vascular p22^phox^ and p47^phox^ expressions, and SOD2 upregulation. Moreover, circulating nitrites and C-reactive protein, increased in SHR, were both reduced in SHR receiving MOI. This was associated to decrease iNOS and NF-*κ*B protein expressions after MOI treatment. In functional studies, the endothelium-dependent carbachol-induced relaxation was improved in MOI-treated SHR resistance arteries. Oral administration of MOI seeds demonstrates vascular antioxidant, anti-inflammatory, and endothelial protective effects in SHR. Our data support the use of MOI seeds in diet against cardiovascular disorders associated with oxidative stress and inflammation such as hypertension, scientifically validating the use of these seeds in Malagasy traditional medicine.

## 1. Introduction

Hypertension is defined as a chronic elevated systolic and/or diastolic blood pressure [1]. High blood pressure is associated with an increased risk of cardiovascular morbidity and mortality and is even identified as the leading cause of avoidable mortality and morbidity worldwide [2, 3]. Until recently, hypertension was mainly associated with developed countries. However, the condition is constantly growing in low- and middle-income countries. Currently, the global prevalence of high blood pressure in adults is around 22% but reaches 30% in Africa where it is the main risk factor of premature death from stroke, cardiac, and renal failure [4]. The health benefits of lowering blood pressure, in particular the resulting reduction in cardiovascular morbidity and mortality, have been indisputably proven [5]. Countries of the African region are thus deploying effective programs and strategies, including traditional medicines, to promote detection, prevention, and control of high blood pressure.

In African countries, in particular in Madagascar, the traditional medicine is a practice based on empiric knowledge of the therapeutic use of plants transmitted from generation to generation. Malagasy medicinal plants are still often used as a first easily affordable resort to treat common disorders including hypertension [6, 7]. For some of these plants, experimental evidence has proven real beneficial effects in the heart, vessels, and hemodynamic parameters. *Cedrelopsis grevei* (Meliaceae) has been shown to have antihypertensive properties via the improvement of endothelial function [8], and, most recently, *Mimosa pigra* (Fabaceae) has been reported to have protective vascular effect via its antioxidant properties in hypoxic rats developing pulmonary arterial hypertension [9].

*Moringa oleifera* (MOI) (Moringaceae) is a tree currently used by traditional medicine in tropical Africa, America, and Asia to treat systemic arterial hypertension [10].

The therapeutic but empirical use of medicinal plants in Madagascar is generally not supported by rational clinical or experimental data. Malagasy population employs MOI seeds against hypertension empirically, and in our previous study, we provided evidence of the cardiac protective role of a treatment with MOI seeds in spontaneously hypertensive rats (SHR) [11]. A physicochemical study suggested antioxidant properties of MOI seed oil [12]. MOI seed oil showed a concentration-dependent free radical scavenging activity probably due to the presence of molecules known to have antioxidant activities such as phenols and in particular flavonoids [12].

The aim of the present study was to assess the in vivo pharmacological efficacy of a diet containing MOI seeds to reduce oxidative and nitrosative stresses and vascular inflammation in SHR. Our results revealed antioxidant and anti-inflammatory effects supporting the use of MOI seeds in diet to improve cardiovascular disorders associated with vascular oxidative stress and inflammation such as hypertension.

## 2. Materials and Methods

### 2.1. Animals

Sixteen-week-old male Wistar Kyoto rats (WKY) and SHR were used. Rats have been fed with either normal food (WKY and SHR groups) or food containing MOI seed powder (750 mg/day/rat; SHR MOI group) for 20 weeks. At 36 weeks of age, rats of the three groups were anesthetized by isoflurane inhalation to harvest blood samples for circulating free 8-isoprostane, nitrite, and C-reactive protein (CRP) measurements. Rats were then sacrificed; the thoracic aorta and mesenteric arteries were then collected for Western blot, immunohistological, and vascular reactivity analyses. All experiments were conducted in agreement with international guidelines for care and use of laboratory animals and approved by our Ethical Committee (authorisation number 00909.01).

### 2.2. Staining and Confocal Microscopy Imaging

Frozen sections of aorta (7 *μ*m thick) on glass slides were used to the in situ detection of superoxide anion (O_2_^−^) with the oxidative fluorescent dye dihydroethidine (DHE, Sigma-Aldrich) as previously described by Miller et al. [13]. DHE oxidizes to EtBr in the presence of O_2_^−^ and shows a red fluorescence.

In another set of experiments, the vessel frozen sections were fixed with cold 100% methanol and incubated (2 h at room temperature) in blocking buffer (5% nonfat dry milk in PBS). Tissue sections were then incubated overnight (4°C) with either a mouse monoclonal antibody antinitrotyrosine (1 : 100, Upstate Biothechnology), a monoclonal murine anti-iNOS (1 : 50, BD Transduction Laboratories), and a polyclonal NF-*κ*B p65 antibody (1 : 100, Cell Signaling), for protein nitrotyrosine, iNOS, or NF-*κ*B p65 immunostaining, respectively. Three washes were followed by incubation (1 h, at room temperature, in the dark) with Alexa fluor-647 anti-mouse or anti-rabbit secondary antibody (1 : 500, Molecular Probes). A Nikon A1-RS inverted laser scanning confocal microscope was used for the optical sectioning of the tissue. Digital image recording was performed using the NIS element software. Images were analyzed and processed by Fiji software.

### 2.3. ELISA Measurements

Plasma measurements of free 8-isoprostane and CRP were performed using ELISA assay kits from Cayman Chemical and Millipore, respectively.

### 2.4. NO Measurements

Circulating NO was determined from nitrite concentration (NO_2_^−^) by a colorimetric assay according to the Griess reaction method (Griess Reagent Kit, Molecular Probes; Invitrogen).

### 2.5. Western Blot Analysis

Aortas were homogenized and lysed. Proteins (50–75 *μ*g) were separated on 12 or 14% SDS-PAGE electrophoresis gel, transferred to nitrocellulose membrane then probed with antibodies to p22^phox^, p47^phox^, gp91^phox^, p67^phox^, SOD1, or SOD2 (Santa Cruz Biotechnology, INC). A monoclonal anti-*α*-tubulin antibody (1 : 5000, Sigma-Aldrich) was used to check protein gel loading and to normalize protein expression. Immunoreactive bands were revealed with a secondary peroxidase-conjugated anti-mouse or anti-rabbit IgG (1 : 5000, Beckman Coulter), detected by enhanced chemiluminescence system (ECL Plus, Amersham Biosciences) and quantified by densitometry.

### 2.6. Arterial Reactivity

Aortas and first branches of superior mesenteric arteries were collected in physiological saline solution (in mM: 130 NaCl, 5.6 KCl, 1 MgCl_2_, 2 CaCl_2_, 11 glucose, 10 Tris, pH 7.4 with HCl), cleaned, and cut in 2 mm long rings. Arterial rings were then mounted on multichannel isometric myograph, bathed in Krebs-Henseleit solution at 37°C, bubbled with 95% O_2_–5% CO_2_, and connected to a force transducer (Pioden controls Ltd, Canterbury, UK for aortic rings; Danish Myo Technology; Aarhus, Danemark, for mesenteric artery rings). After equilibration, the contractile response to KCl 60 mM was measured. Endothelial function was assessed by measuring the relaxing response to cumulative doses of carbachol (CCh, 1 nM −10 *μ*M) of rings precontracted by phenylephrine (PhE, 1 *μ*M). Digital data were recorded by a MacLab/4e recorder and analyzed using a LabChart v7 software (AD Instruments, Paris, France).

### 2.7. Data Analysis

A one-way ANOVA with subsequent Bonferroni post hoc test or the ANOVA on ranks were performed for all the experiments except for them of vascular reactivity for which we used a two-way analysis of variance for repeated measures with subsequent Bonferroni post hoc test. All statistical analyses were realized with Statview software (SAS Institute, Cary, NC, USA). All values are presented as mean ± standard deviation for *n* experiments, *n* representing the number of rat samples. ^∗^*p* < 0.05 was considered to be statistically significant.

## 3. Results

### 3.1. MOI Reduces Oxidative Stress in SHR Aortas

**Figure 1:**
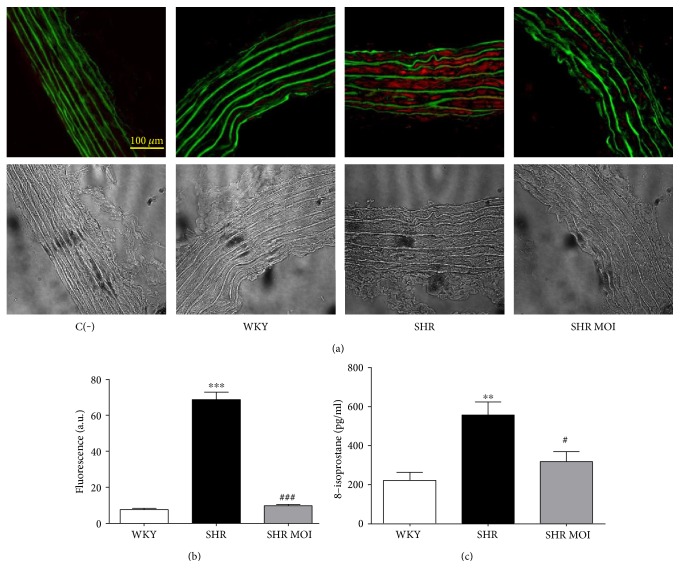
(a) Oxidative stress (red staining by DHE of O_2_^−^ in rat aortas from Wistar Kyoto (WKY), SHR untreated (SHR), and SHR treated with MOI (SHR MOI)). Green fluorescence corresponds to autofluorescence of elastin. Below phase-contrast images. Negative control C(−) without DHE. (b) Fluorescence is expressed in arbitrary units (a. u.), WKY (white plot), SHR (black plot), and SHR MOI (grey plot) (*n* = 5 for each group, ^∗∗∗^*p* < 0.001 SHR versus WKY and ^###^*p* < 0.001 SHR MOI versus SHR). (c) Circulating levels of free 8-isoprostane expressed in pg/ml of plasma (*n* = 6‐7 for each group, ^∗∗^*p* < 0.01 SHR versus WKY and ^#^*p* < 0.05 SHR MOI versus SHR).

To assess the effect of MOI treatment on oxidative stress characterizing hypertensive rats, we directly assessed the in situ production and the topographical distribution of O_2_^−^ in aortic sections from WKY, SHR, and SHR MOI. As expected, compared to control WKY aortas, SHR aortas displayed a marked increase in EtBr fluorescence, reflecting elevated oxidative stress in the vascular wall (Figures 1(a) and 1(b)). In contrast, the O_2_^−^ staining in aortic sections from SHR MOI was comparable to that from WKY rats (Figures 1(a) and 1(b)).

The antioxidant effect of MOI was further addressed by the measurement of free circulating 8-isoprostane, a plasmatic oxidative stress marker. In SHR, the plasmatic level of 8-isoprostane was significantly increased compared to WKY (Figure 1(c)). MOI treatment in SHR significantly reduced the plasma concentration of 8-isoprostane to a level similar to that observed in WKY (Figure 1(c)). These data are consistent with a vascular and systemic antioxidant effect of MOI treatment.

### 3.2. MOI Reduces NADPH Oxidase Expression and Upregulates SOD2

**Figure 2:**
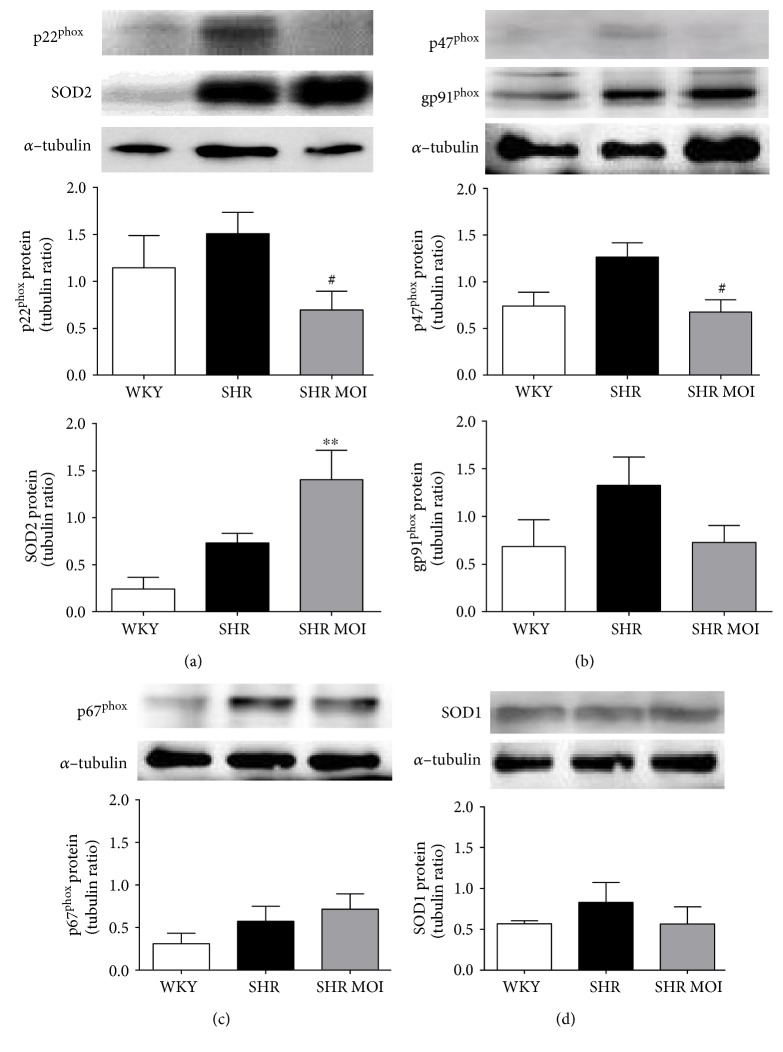
Representative Western blot and corresponding densitometric analysis of NADPH oxidase subunits (a–c) and SOD isoformes (a and d) in aortas from WKY rats (white plot), untreated SHR (SHR, black plot), and MOI-treated SHR (SHR MOI, grey plot). (a) p22^phox^ (*n* = 4, ^#^*p* < 0.05, SHR MOI versus SHR) and SOD2 protein expression (*n* = 4, ^∗∗^*p* < 0.01, SHR MOI versus WKY). (b) p47^phox^ and gp91^phox^ protein expression (*n* = 4 − 6, ^#^*p* < 0.05, SHR MOI versus SHR or any significant difference, resp.). (c and d) p67^phox^ and SOD1 protein expression, respectively (*n* = 4, any significant difference). Data were normalized to *α*-tubulin densitometry.

The NADPH oxidase (NOX) family of enzymes is a major source of ROS in the cardiovascular system. We thus next analyzed the effect of MOI on the aortic expression of four subunits of the active NOX2 enzyme complex. The expression of the catalytic subunit NOX2 (gp91^phox^) and the expression of the regulatory subunits p22^phox^, p47^phox^, and p67^phox^ tended to be higher in SHR than in WKY, although the differences did not reach significance (Figures 2(a), 2(b), and 2(c)). MOI treatment in SHR significantly decreased p22^phox^ and p47^phox^ expressions (Figures 2(a) and 2(b)). Regarding the antioxidant enzyme superoxide dismutase (SOD), SOD1 was expressed similarly in WKY, SHR, and SHR MOI (Figure 2(d)). The mitochondrial isoform of SOD, SOD2, equally expressed in WKY and SHR, was significantly increased in SHR MOI, suggesting that MOI treatment might improve arterial mitochondrial antioxidant activity (Figure 2(a)).

### 3.3. MOI Reduces Nitrotyrosine Expression in Rat Aortas

**Figure 3:**
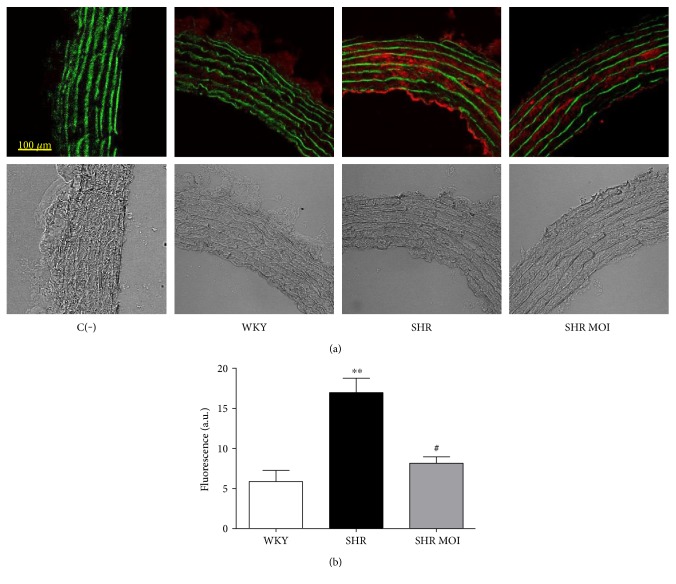
(a) Nitrosative stress (red staining), nitrotyrosine of proteins, in rat aortas from Wistar Kyoto (WKY), untreated SHR (SHR), and SHR treated with MOI (SHR MOI). Green fluorescence corresponds to autofluorescence of elastin. Below phase-contrast images. Negative control C(−), incubated only with the secondary murine fluorescent-labeled antibody, displays any specific red staining but only the green fluorescence of elastin. (b) Red fluorescence is expressed in arbitrary units (a.u.), (*n* = 4, ^∗∗^*p* < 0.01 SHR versus WKY and ^#^*p* < 0.05 SHR MOI versus SHR).

Oxidative stress is generally associated with nitrosative stress due to the overproduction of NO of inflammatory origin and its interaction with superoxide anion to form the reactive peroxynitrite anion (ONOO^−^). Although there are multiple targets for the reactive ONOO^−^, the presence of 3-nitrotyrosine, whether free or protein bound, has been proposed as a marker of ONOO^−^ formation. To assess the effect of MOI on nitrosative stress, we thus performed immunostaining of nitrotyrosine in aorta sections. A strong staining was observed in both endothelium and the medial layer of SHR aortas compared to WKY (Figures 3(a) and 3(b)). In aortas from SHR MOI, nitrotyrosine staining was reduced to a level similar to that of WKY aortas (Figures 3(a) and 3(b)), indicating that MOI treatment decreased the enhanced nitrosative stress in hypertensive rats.

### 3.4. MOI Reduces iNOS Expression in Rat Aortas

**Figure 4:**
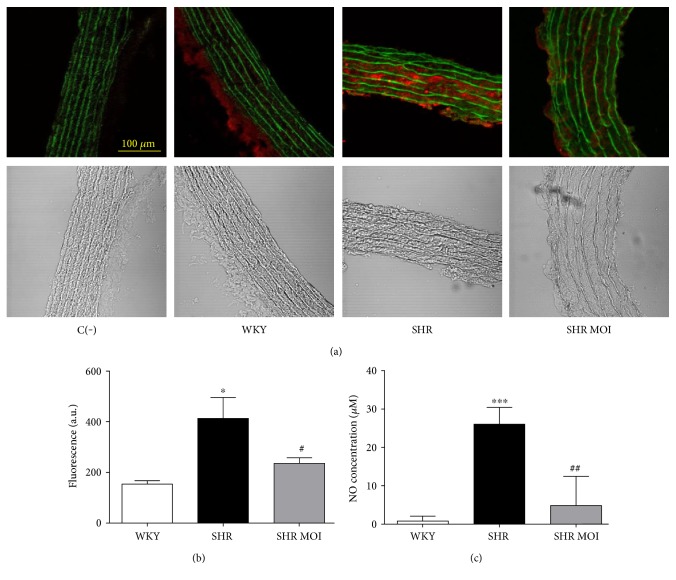
(a) Red staining of iNOS observed in rat aortas from Wistar Kyoto (WKY), untreated SHR (SHR), and SHR treated with MOI (SHR MOI). Green fluorescence corresponds to autofluorescence of elastin. Below phase-contrast images. Negative control C(−), incubated only with the secondary murine fluorescent-labeled antibody, displays any red staining but only the green fluorescence of elastin. (b) Red fluorescence is expressed in arbitrary units (a.u.), (*n* = 4, ^∗^*p* < 0.05 SHR versus WKY and ^#^*p* < 0.05 SHR MOI versus SHR). (c) Plasmatic levels of nitrites expressed in *μ*M (*n* = 5 − 6, ^∗∗∗^*p* < 0.001 SHR versus WKY and ^##^*p* < 0.01 SHR MOI versus SHR).

To evaluate the anti-inflammatory properties of MOI treatment, we first analyze the expression of iNOS by immunostaining on aortic sections. No or weak red staining of iNOS was found in control WKY aortic sections while a marked staining was observed in both endothelium and the medial layer of SHR arteries, indicating upregulation of iNOS (Figures 4(a) and 4(b)). MOI treatment significantly reduced iNOS expression in SHR (Figures 4(a) and 4(b)). Assessment of circulating NO through measurement of plasma nitrite levels confirmed this observation, showing a strong increase of plasmatic nitrites in SHR group, which was significantly decreased in the SHR MOI group (Figure 4(c)).

### 3.5. MOI Reduces NF*κ*B p65 Expression/Activation in Rat Aortas

**Figure 5:**
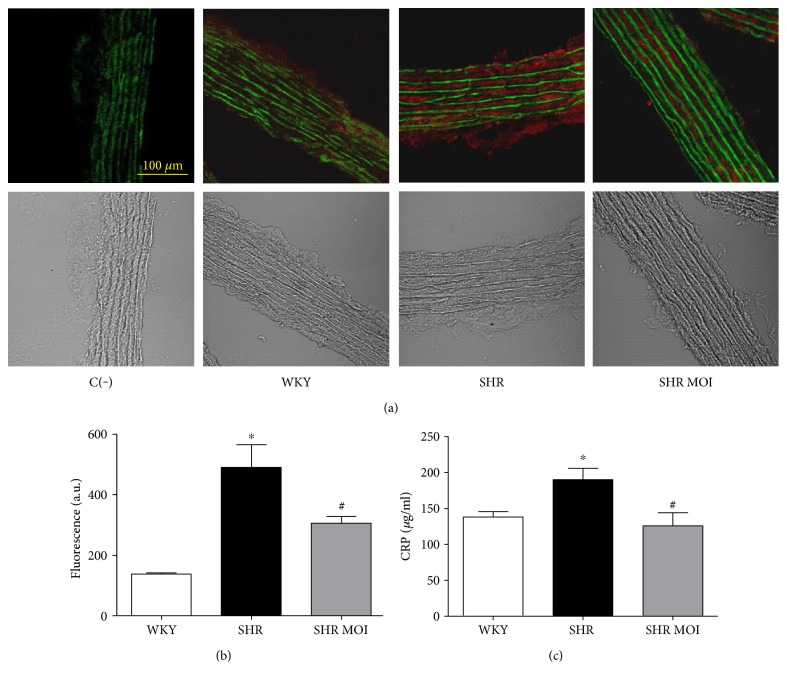
(a) Immunohistochemical red staining of the p65/RelA subunit of NF*κ*B in rat aortas from Wistar Kyoto (WKY), SHR untreated (SHR), and SHR treated with MOI (SHR MOI). Green fluorescence corresponds to autofluorescence of elastin. Below phase-contrast images. Negative control C(−), incubated only with the secondary rabbit fluorescent-labeled antibody, displays any red staining but only the green fluorescence of elastin. (b) Red fluorescence is expressed in arbitrary units (a.u.) (*n* = 3 − 4, ^∗^*p* < 0.05 SHR versus WKY and ^#^*p* < 0.05 SHR MOI versus SHR). (c) Circulating level of C-reactive protein (CRP) (*n* = 5 − 7, ^∗^*p* < 0.05 SHR versus WKY and ^#^*p* < 0.05 SHR MOI versus SHR).

The iNOS expression is induced, at least in part, through the activation of NF*κ*B p65. We thus investigated the expression/activation of this transcription factor by immunostaining of p65 in aorta sections. The negative control obtained by incubation with the secondary murine fluorescent-labeled antibody did not display any red staining but only the green autofluorescence of the elastin (Figure 5(a)). A weak red staining of NF*κ*B p65 was found in control WKY vessels while a marked staining was observed in the vascular wall of SHR aortas (Figures 5(a) and 5(b)). MOI treatment significantly reduced the NF*κ*B p65 red staining (Figures 5(a) and 5(b)). This beneficial anti-inflammatory effect of MOI seeds was confirmed by the measurement of plasmatic CRP (Figure 5(c)). CRP level, significantly increased in SHR, was reduced to a level similar to that of WKY by MOI (Figure 5(c)). These data confirm vascular and systemic anti-inflammatory properties of MOI seeds.

### 3.6. MOI Ameliorates Endothelial Function in Mesenteric Arteries

**Figure 6:**
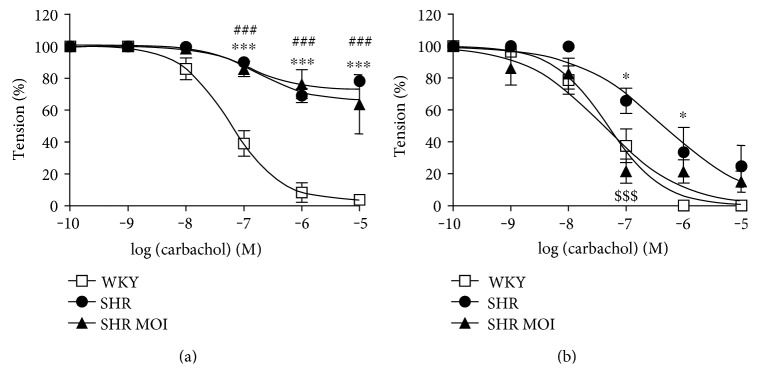
(a) Aorta and (b) mesenteric artery relaxation to cumulative doses of carbachol (^∗^*p* < 0.05 and ^∗∗∗^*p* < 0.001 WKY versus SHR; ^###^*p* < 0.001 WKY versus SHR MOI; ^$$$^*p* < 0.001 SHR versus SHR MOI, *n* = 5 for each group of vessels, values are expressed as mean ± S.E.M for each dose of carbachol).

To test if MOI treatment was able to ameliorate vascular function in hypertensive rats, endothelial function was assessed in both aortic and mesenteric vessels. We found similar reduced endothelial-dependent relaxation to cumulative doses of carbachol in both SHR and SHR MOI aortas compared to WKY aortas, suggesting that MOI was not able to ameliorate endothelial function in conductance arteries (Figure 6(a)). However, MOI restored the altered carbachol-induced endothelial-dependent relaxation in resistance SHR mesenteric arteries (pEC50: 7.31 in WKY and 7.38 in SHR MOI versus 6.3 in SHR; Figure 6(b)).

## 4. Discussion

Our results provide evidence for beneficial vascular and systemic antioxidant and anti-inflammatory properties of MOI seeds in hypertension. Orally administrated, MOI seed powder limits the vascular oxidative and nitrosative stresses in aortas and improves the endothelial function of SHR resistance arteries.

High blood pressure is associated with increased reactive oxygen species (ROS) formation in vessels and target organs such as the brain and kidney [14]. ROS increase vascular dysfunction by promoting inflammation, thus establishing a positive feedback mechanism that participates to the development of hypertension [15]. O_2_^−^, essentially of NOX origin, reduces endothelial NO bioavailability and promotes nitrosative stress leading to endothelial dysfunction and vascular tone increase that also contributes to hypertension [16–18]. SHR is a genetic model that exhibits many features of the human idiopathic hypertension. Indeed, hypertension in SHR follows the same progression as in humans, with prehypertensive, developing, and sustained hypertensive phases, each phase lasting at least several weeks [19, 20]. Vascular oxidative stress precedes the increase in blood pressure and peripheral resistance in SHR [21, 22], and administration of the antioxidant tempol prevents age-related development of hypertension in SHR, suggesting the therapeutic interest of targeting oxidative stress [23].

SHR is known to exhibit increased NOX-driven O_2_^−^ generation in resistance and conducting arteries associated with NOX subunit overexpression and enhanced oxidase activity [24, 25]. NOX-mediated O_2_^−^ production has also been observed in the vascular wall of patients with hypertension and atherosclerosis [26]. Here, we show that MOI reduced the expression of NOX regulatory subunits in SHR, suggesting that its antioxidant effect results, at least in part, from the downregulation of NOX subunits and the subsequent reduction of NOX-driven O_2_^−^ generation. We cannot exclude that MOI-induced decrease in NOX-dependent O_2_^−^ generation might also indirectly result from the reduced level of circulating free 8-isoprostane in MOI-treated SHR. 8-isoprostane is not only a marker of oxidative stress but it also increases, itself, oxidative stress by enhancing NOX expression and activity, thus creating a self-perpetuating positive feedback loop [27]. By leading to a reduction of this positive feedback mechanism, the MOI-induced reduction of free 8-isoprostane can thus participate to the decrease of NOX-driven O_2_^−^ generation.

Furthermore, we found that MOI treatment in SHR also increases the aortic expression of SOD2, a mitochondrial Mn-containing enzyme described as one of the most important protective systems against oxidative stress [28]. SOD2 downregulation is proportionally linked to increased cell apoptosis under stress conditions while SOD2 upregulation reduces cell death and ROS levels during oxidative stress [29]. SOD2 upregulation is associated with the improvement of endothelial function in experimental models of diabetes, hypertension, and metabolic syndrome [30, 31]. Thus, the antioxidant action of MOI is likely due to both a reduction of O_2_^−^ production by the downregulation of p22^phox^ and p47^phox^ and the increased O_2_^−^ elimination through SOD2 upregulation. Such a stimulation of antioxidant mechanism has been previously described for another Malagasy plant, *Cedrelopsis grevei*, used as an antiherpertensive treatment. This plant upregulated another SOD, the Cu/Zn SOD, and improved endothelial function in rats [8].

Oxidative stress is mainly associated with inflammation and NO overproduction by iNOS upregulation through the activity of transcription factor NF*κ*B p65 [32]. NO reacts with O_2_^−^, leading to peroxynitrite production, subsequent protein tyrosine nitration, and cell toxicity [17]. In agreement with the antioxidant effect of MOI, we observed a reduction of nitrosative stress in aortas from SHR MOI associated with a reduction of NF*κ*B p65 expression/activation and iNOS expression, thus revealing an anti-inflammatory action of MOI. A systemic anti-inflammatory effect of MOI treatment was confirmed by the low circulating levels of CRP in SHR MOI compared to SHR.

Functionally, we found that MOI treatment improves endothelial relaxation in resistance arteries in SHR. Oxidative stress is known to be causally involved in the endothelial dysfunction associated with hypertension [16–18]. Our results thus further support the beneficial effect of antioxidants on endothelial function in hypertensive condition and the therapeutic interest of plants such as MOI that target oxidative stress.

The complete composition of MOI seeds has been previously described [33, 34]. The beneficial vascular effects observed in the present work are probably due to the presence in the seeds of tocopherols, phenolic acids (gallic and ferulic acids), and flavonoids (such as quercetin, catechin, and epicathechin) known for their potential antioxidant activity [34]. Moreover, we previously identified glucosinolates, in particular glucomoringin, in MOI seeds extract that can also contribute to its cardiovascular protective role [11]. Glucomoringin hydrolysis by myrosinase activity during food processing or digestion leads to the formation of isothiocianates (ITCs) [35]. Myrosinase is an enzyme acting during vegetal cell lysis and death [36]. It was previously demonstrated that the ITCs produced from glucosinolates of *Brassicaceae* inhibited NF-*κ*B and prevented oxidative stress in several oxidative and inflammatory diseases [37, 38]. Furthermore, glucoeurocin-derived ITCs contained in rocket were shown to have indirect antioxidant activity by inducing phase-II enzymes that play an important role in the detoxification of electrophiles and subsequent free radical level reduction [39]. More recently, the anti-inflammatory and antioxidant activity of the glucomoringin-derived ITC moringin has been described in LPS-stimulated macrophages when associated with cannabidiol [40]. In agreement with these previous findings, glucomoringin ITCs could thus participate, in association with other compounds such as tocopherols and flavonoids, in the in vivo antioxidant and anti-inflammatory cardiovascular effects evidenced in MOI SHR. The beneficial effects of whole MOI seeds are thus likely due to their original composition and the synergistic action of several antioxidant compounds rather to a unique active compound.

## 5. Conclusions

In conclusion, the present study demonstrates the antioxidant and anti-inflammatory effect of orally administrated MOI seeds and their beneficial action on the endothelial function in a rat model of hypertension. The present study thus supports scientifically the empirical use of MOI in traditional Malagasy medicine to treat high blood pressure and other cardiovascular disorders associated with inflammation and oxidative stress.

## Abbreviations

CRP: C-reactive protein
DHE: Dihydroethidine
iNOS: Inducible NO synthase
ITCs: Isothiocyanates
MOI: *Moringa oleifera*
NO: Nitric oxide
NO^−^_2_: Nitrite
O^−^_2_: Superoxide anion
ONOO^−^: Peroxynitrite
SHR: Spontaneously hypertensive rats
SHR MOI: SHR treated with *Moringa oleifera* seeds
SOD: Superoxide dismutase
WKY: Wistar Kyoto rats.
